# Concurrent neuromechanical and functional gains following upper-extremity power training post-stroke

**DOI:** 10.1186/1743-0003-10-1

**Published:** 2013-01-21

**Authors:** Carolynn Patten, Elizabeth G Condliffe, Christine A Dairaghi, Peter S Lum

**Affiliations:** 1 Brain Rehabilitation R&D Center (151A), Malcolm Randall VA Medical Center, 1601 SW Archer Rd, Gainesville, FL, 32608, USA; 2 Department of Physical Therapy, University of Florida, Gainesville, FL, USA; 3 Division of Physical Medicine and Rehabilitation, University of Alberta, Edmonton, AB, Canada; 4 Department of Biomedical Engineering, University of Alberta, Edmonton, AB, Canada; 5 Rehabilitation Research Center, VA Palo Alto Health Care System, Palo Alto, CA, USA; 6 Department of Biomedical Engineering, The Catholic University of America, Washington, DC, USA; 7 Veterans Affairs Medical Center, Washington, DC, USA; 8 Center for Applied Biomechanics and Rehabilitation Research, National Rehabilitation Hospital, Washington, DC, USA

**Keywords:** Stroke, Recovery, Function, Upper-extremity, EMG, Strength(ening), Hemiparesis, Hypertonia, Muscle strength, Rehabilitation

## Abstract

**Background:**

Repetitive task practice is argued to drive neural plasticity following stroke. However, current evidence reveals that hemiparetic weakness impairs the capacity to perform, and practice, movements appropriately. Here we investigated how power training (i.e., high-intensity, dynamic resistance training) affects recovery of upper-extremity motor function post-stroke. We hypothesized that power training, as a component of upper-extremity rehabilitation, would promote greater functional gains than functional task practice without deleterious consequences.

**Method:**

Nineteen chronic hemiparetic individuals were studied using a crossover design. All participants received both functional task practice (FTP) and HYBRID (combined FTP and power training) in random order. Blinded evaluations performed at baseline, following each intervention block and 6-months post-intervention included: Wolf Motor Function Test (WMFT-FAS, Primary Outcome), upper-extremity Fugl-Meyer Motor Assessment, Ashworth Scale, and Functional Independence Measure. Neuromechanical function was evaluated using isometric and dynamic joint torques and concurrent agonist EMG. Biceps stretch reflex responses were evaluated using passive elbow stretches ranging from 60 to 180º/s and determining: EMG onset position threshold, burst duration, burst intensity and passive torque at each speed.

**Results:**

Primary outcome: Improvements in WMFT-FAS were significantly greater following HYBRID vs. FTP (*p* = .049), regardless of treatment order. These functional improvements were retained 6-months post-intervention (*p* = .03).

Secondary outcomes: A greater proportion of participants achieved minimally important differences (MID) following HYBRID vs. FTP (*p* = .03). MIDs were retained 6-months post-intervention. Ashworth scores were unchanged (*p* > .05).

Increased maximal isometric joint torque, agonist EMG and peak power were significantly greater following HYBRID vs. FTP (*p* < .05) and effects were retained 6-months post-intervention (*p*’s < .05). EMG position threshold and burst duration were significantly reduced at fast speeds (≥120º/s) (*p*’s < 0.05) and passive torque was reduced post-washout (*p* < .05) following HYBRID.

**Conclusions:**

Functional and neuromechanical gains were greater following HYBRID vs. FPT. Improved stretch reflex modulation and increased neuromuscular activation indicate potent neural adaptations. Importantly, no deleterious consequences, including exacerbation of spasticity or musculoskeletal complaints, were associated with HYBRID. These results contribute to an evolving body of contemporary evidence regarding the efficacy of high-intensity training in neurorehabilitation and the physiological mechanisms that mediate neural recovery.

## Background

Upper-extremity hemiparesis is among the most significant and persistent physical disabilities following stroke and represents a critical barrier to independence [1]. While the problem is well recognized, there is little evidence demonstrating the most effective approach for promoting functional motor recovery of the hemiparetic upper-extremity [2].

Prominent manifestations of compromised motor control following stroke include: impaired inter-segmental coordination [3], hyperreflexia or spasticity [4], and weakness [5]. Rather than mechanical factors such as muscle fibre type or cross-sectional area, hemiparetic weakness results predominantly from disorganized neuromotor output, including impaired descending motor drive, and activation impairment [6,7]. Accumulating evidence suggests that weakness plays a more significant role than traditionally believed and contributes directly to compromised motor function post-stroke [8-10]. In contrast to fundamental traditional clinical tenets [11], contemporary research demonstrates that neither high-exertion activities nor resistance training, per se, exacerbate spasticity [12-16]. Lower extremity resistance exercise has revealed improvements in functional task performance including: walking, rising from a chair, and stair climbing [10,17-19] and self-perceived disability [20] in persons post-stroke. However, the role of strength [8,9] and the effects of strengthening have only recently been systematically investigated in the hemiparetic upper-extremity [21-23].

Here we investigated two forms of upper-extremity rehabilitation for persons post-stroke: 1) functional task practice (FTP) and 2) functional task practice combined with upper-extremity power training (i.e., dynamic high-intensity resistance training) which we refer to as HYBRID. We hypothesized that inclusion of power training in upper-extremity rehabilitation would produce greater effects on clinical and neuromechanical indicators of functional motor recovery without producing detrimental effects including exacerbation of spasticity. Because there is little evidence to support inclusion of either high-intensity or resistance training, we conducted a clinical trial to investigate the feasibility, safety and efficacy of upper-extremity power training in persons post-stroke. Our observations confirm our hypothesis and demonstrate positive functional outcomes, increased strength and joint power, improved reflex modulation and retention of treatment effects in the absence of additional intervention. Importantly, our findings indicate no negative consequences (i.e., exacerbation of spasticity, joint pain or injury) resulting from inclusion of power training in upper-extremity rehabilitation.

## Methods

### Participants

We studied nineteen individuals in the chronic phase of recovery, operationally defined as 7-18 months post-stroke. All participants had completed directly supervised medical rehabilitation programs and agreed to maintain participation in community-based physical activities (e.g., adaptive physical education, support groups, individual work with a personal trainer, etc.) constant through the full period of study including a 6-month retention interval. Compliance with this agreement was monitored through activity logs kept by participants (and their spouses/caregivers), which were returned at each evaluation session and reviewed by the Principal Investigator and study personnel.

Inclusion criteria for participation were: i) clinical presentation of a single, unilateral stroke; ii) ability to produce active, volitional movement out of the plane of gravity at the shoulder and elbow; iii) demonstration of at least 10º of active wrist extension, 10º active thumb abduction, and 10º active extension of any two digits, three times within one minute; iv) freedom from significant upper extremity joint pain, range of motion limitations, and/or sensory deficits as revealed by clinical examination [24]. The Neurobehavioral Cognitive Status Exam (“Cognistat”) [25] was administered to determine participants’ abilities to comprehend, provide decisional consent, learn and follow three step commands. Diagnosis of stroke, including mechanism and location was confirmed by review of medical records, radiology reports and documentation by the participant’s referring physician. Participants were recruited from the sponsoring institution and the greater community, which facilitated enrollment of a demographically representative participant sample. All procedures were approved by the Stanford University Panels on Human Subjects in Research. Written, informed consent was provided by all participants prior to enrollment, randomization and involvement in study activities.

### Study design

**Figure 1:**
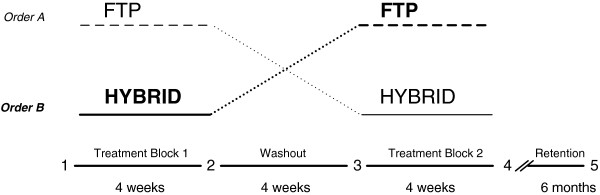
**Cross**-**over research design.** All participants received both FTP (control) and HYBRID (experimental) treatments, randomized to order. Order A received FTP first, followed by a washout period, and then participated in the HYBRID treatment. Order B (highlighted in bold) received the HYBRID first followed by the washout and then FTP. Treatment blocks were each 4 weeks separated by a 4-week washout period. Evaluations were conducted at baseline (1), following the first treatment block (2), following the washout period (3), following the second treatment block (4) and following a 6-month no treatment retention period (5).

The study involved a randomized, double-blind crossover design [26]. All participants received both the control (FTP) and experimental (HYBRID) interventions, randomized to treatment order (Figure 1). Treatment Order A was operationally defined as FTP followed by HYBRID and Treatment Order B as HYBRID followed by FTP. Treatment was delivered in two 4-week blocks of twelve sessions each, interspersed with a 4-week washout period. Thus, each participant received a total of 24 sessions of one-on-one treatment with a physical therapist over a 12-week period. All participants were treated by the same physical therapist. Blinded evaluators conducted clinical and neuromechanical assessments at: baseline, following each block of therapy, following the washout period, and again at 6-months post-intervention.

### Study population

**Figure 2:**
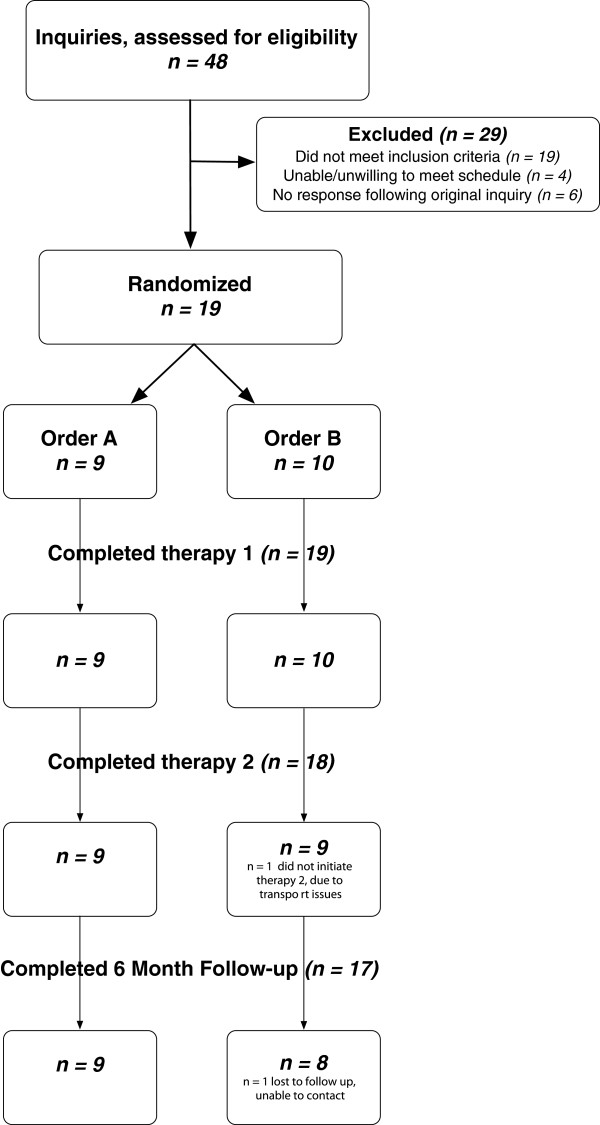
**Consort Diagram.** Flow of participants through all stages of the study.

Of the 48 persons who inquired regarding study participation, 23 met eligibility criteria. Nineteen persons agreed to enrollment and were randomized. The flow of study participants through all stages of the study is depicted in Figure 2. Participant characteristics, demographics and baseline clinical metrics are reported in Table 1.

**Table 1:** Participant demographics

**Characteristic**	**Order A**	**Order B**
	**n = 9**	**n = 10**
Mean age, yr (±SD)	64.7 (9.7)	72.9 (11.1)
Gender	6 M, 3 F	9 M, 1 F
Time since onset, mo	14.7 (2.7)	11.4 (4.3)
Side affected	5 L, 4 R	5 L, 5 R
Mechanism of Stroke		
Ischemic	7	7
Hemorrhagic	2	2
Infarct w/hemorrhagic conversion		1
Lesion location		
Cortical	6	3
Subcortical	3	2
Not specified		5
Upper-extremity Fugl-Meyer Score	37.3 (13.1)	43.2 (10.6)
(total 66 points)		
Fugl-Meyer Shoulder-Elbow Score	18.9 (5.4)	20.4 (6.0)
(total, 30 points)		
Ashworth Score (shoulder + elbow)	3.9 (1.5)	3.4 (2.0)
(total 8 points)		
Wolf Motor Function Test	2.9 (1.1)	3.1 (0.8)
FAS (range 0–5)		
Functional Independence Measure	84.3 (5.0)	76.4 (14.1)
(Total 91 points)		

Diagnosis of stroke and lesion location were confirmed via review of the participant’s medical records. Data presented are mean (±SD).

### Randomization and blinding

The shoulder-elbow portion (30 points) of upper-extremity Fugl-Meyer motor score [27] was used to classify participants as higher (≥20 points) and lower (<20 points) functioning. Separate random orders prepared at study initiation for higher and lower functioning participants were allocated to sealed envelopes and kept by the study coordinator in a locked drawer. Following baseline clinical assessment, the blinded evaluator informed the study coordinator of the participant’s hemiparetic severity (i.e., higher v. lower). The coordinator selected a sequentially numbered sealed envelope from the appropriate group (i.e., higher vs. lower). This envelope was given to the treating physical therapist who broke the seal to reveal the assignment to treatment order. Stratification on the basis of hemiparetic severity was done to assure baseline equivalence between groups (i.e., Order A and Order B). Participants were informed that the study goal was to investigate the efficacy of two forms of upper-extremity rehabilitation and were actively counseled to not discuss the specific therapeutic activities with study personnel other than the treatment physical therapist.

### Therapeutic interventions

Algorithms for both the FTP and HYBRID interventions have been described in detail elsewhere [21]. Briefly, treatments were administered on alternate days (i.e., Monday, Wednesday, Friday) at the same time of day. Individual sessions were 75 minutes in duration and initiated with 10-15 minutes of stretching and passive range of motion.

#### Functional task practice

**Figure 3:**
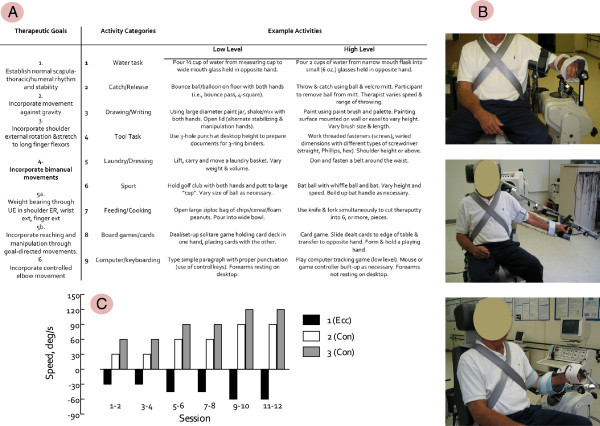
**Therapeutic interventions.** The therapeutic interventions used in this study included functional task practice (FTP) and upper-extremity power training combined with FTP (HYBRID). The structure of the FTP program is outlined in **Panel A** with examples of how activities were identified for study participants of varying abilities and progressed over the course of the intervention. Power training was delivered using a commercially available dynamometer fitted with custom attachments to enable non-standard positioning and accommodate individuals with impaired grasp. Pictured in **Panel B** are configurations for elbow flexion/extension (top), shoulder abduction (middle) and shoulder external rotation. The elbow flexion/extension configuration was also used for stretch reflex testing. Power training involved 3 sets of 10 repetitions of each exercise: shoulder flexion, shoulder abduction, shoulder external rotation, elbow flexion/extension. The criterion speeds for each set were varied using the protocol illustrated in **Panel C**. The first set of each exercise involved eccentric actions in which the participant resisted the dynamometer through the full range of motion. Using data reported by Colsen et al [30] (see Figure 1) to estimate the power produced (i.e., torque x velocity) per contraction, the program was systematically progressed by increasing workload by 44% (Sessions 5-8 relative to 1-4) and 84% (Sessions 9-12 relative to 1-4).

The control intervention involved functional task practice structured according to principles of motor learning [28] and utilized a progression of six therapeutic goals and nine activity categories. Specific tasks, chosen from the activity categories, were practiced on a structured rotation within the framework of the current therapeutic goal. Each of the six treatment goals was addressed for two sessions and treatment progressed to the next therapeutic goal independent of whether mastery of the current goal had been achieved. A variety of therapeutic tasks were developed for each of the nine activity categories (Figure 3), which were identified for individual participants on the basis of functional level, his/her personal goals and needs. Within each session the time devoted to each activity category was held constant at 10 minutes. Thus, individual sessions involved tasks from six activity categories. Each of the nine activity categories was addressed twice per week. Our approach: i) allowed for structure and repeatability across multiple participants in a three-year intervention study, ii) afforded flexibility to accommodate participants presenting with varied hemiparetic severity and functional deficits, and iii) allowed the therapist to tailor intervention using patient-centered goals [29].

#### HYBRID intervention

The experimental intervention combined power training with FTP. Each treatment session divided time between upper-extremity power training (35 minutes) and FTP (25-30 minutes). The abbreviated FTP component addressed six of the nine activity categories, which were selected on the basis of the participant’s abilities and goals. Each individual session involved practice of four activity categories for seven minutes each. Power training involved four reciprocal upper-limb movements: shoulder abduction/adduction, shoulder flexion/extension, shoulder external/internal rotation and transverse plane elbow flexion/extension and was delivered using a Biodex System 3.0 Pro dynamometer^a^. Custom attachments, designed to accommodate hand and wrist weakness, were used to enable hemiparetic participants to engage the dynamometer without grasping and to optimize positioning for performance through a full range of motion for each joint (Figure 3). Where necessary, the attachments were counterbalanced to minimize the effect of lifting the weight of the attachment against gravity. Each power training session involved three sets of 10 repetitions of each movement [31]. The first set was eccentric (e.g., resisting an externally imposed load) and the second two sets were concentric, delivered at different criterion speeds. The dynamometer was controlled in isokinetic mode (i.e., constrained to pre-set speed). Over the course of treatment, movement speeds were advanced in 30º/s increments in concentric sets (i.e., from 30-120º/s) and 15º/s increments in eccentric sets (i.e., from 30-75º/s) (Figure 3). Power training targeted proximal joints (e.g., shoulder and elbow).

### Participant compliance

All treatment sessions were completed within the timeframe of the study design. Several factors specific to our setting enabled 100% compliance with the intervention protocols. First, this study was conducted in a free standing rehabilitation research center where study personnel were tasked to project activities rather than routine clinical care. If a participant was unable to attend a session, makeup sessions were scheduled as soon as possible and, only if necessary, on an adjacent day. This measure was taken to assure delivery of the requisite number of treatments in the timeframe specified by the study protocol. The costs of study personnel and participant transportation were underwritten by grant support, thus the therapeutic intervention was delivered at no cost to the participant or his/her insurance provider. In exchange, participants agreed to all intervention sessions and return for follow up evaluations.

### Assessment battery

A comprehensive battery of clinical and neuromechanical assessments was administered by blinded evaluators at five times across the study: baseline, following each treatment block, following the washout period and at six months post-intervention. Stretch reflex responses were assessed from only the first treatment block and the washout period.

#### Clinical assessment

Clinical outcomes were assessed using tools for which validity and reliability have previously been established in individuals post-stroke. Because the purpose of this investigation was to demonstrate treatment efficacy [32], these focused on assessments representing the body structure/function and activity levels of the International Classification of Functioning, Disability and Health [33] and included: the upper-extremity portion of the Fugl-Meyer motor assessment [27], the Ashworth Scale [34,35] the Wolf Motor Function Test-Functional Abilities Scale (WMFT-FAS) [36-38], and the Functional Independence Measure (FIM) [39]. Self-report questionnaires probing participation and self-efficacy are more appropriately used in later stage clinical investigation of treatment effectiveness [32]. The WMFT-FAS [37,38] served as the primary outcome.

#### Neuromechanical assessment

Joint torques were obtained from the dynamometer during elbow flexion (EF) and extension (EE), shoulder flexion (SF), abduction (S’Abd) and external rotation (S’ER) in the following four conditions: isometric (MVIC), and concentric actions at 30, 75 and 120º/s at each of the five assessments. Neuromotor activation was assessed using surface electromyography recorded from eight upper-extremity muscles (biceps brachii, triceps brachii, anterior/middle/posterior deltoid, infraspinatus, brachioradialis, and pectoralis major) using active, pre-amplified surface electrodes (17mm inter-electrode distance). To mitigate the effects of inter-individual variability of electrode placement, subcutaneous adipose tissue thickness and other sources of variability, EMG electrode were placed using the convention of Delagi [40], referenced to anatomical landmarks, by only one investigator. Analog signals (i.e., torque and position) were sampled directly from the dynamometer concurrently with EMG at 2 kHz using custom-written software and written directly to disk for offline analysis. Reliability of neuromechanical measures in this study population has been established in our laboratory [7,41,42].

#### Stretch reflexes

Stretch reflex responses were elicited using passive ramp-and-hold elbow extensions applied using the dynamometer [43]. The experimental configuration is illustrated in Figure 3 (Panel B, top). Surface EMG was recorded from the brachioradialis, biceps brachii, and triceps brachii (long head) muscles using pre-amplified electrodes^b^ (MA-311). Analog position and torque signals were sampled directly from the dynamometer at 2kHz written directly to disk for offline analysis.

For each test session, participants were seated in the dynamometer chair with the back angled at 85º, the trunk stabilized using waist and trunk straps, and the feet supported using the leg rest. The hemiparetic arm was positioned with the shoulder in 70-80º abduction, and 5-10º forward flexion with the medial epicondyle of the humerus aligned with the dynamometer rotational axis. The arm was stabilized using an adjustable support to balance the weight of the limb and eliminate excess shoulder rotation during elbow flexion and extension. The wrist and hand were positioned in pronation using a pre-fabricated wrist splint and straps added to the standard dynamometer wrist attachment. Passive elbow extensions covered a 100º range ending at the participant’s full anatomical range of motion. The anatomical position was determined using a handheld goniometer and reported in degrees of elbow flexion (i.e., full extension = 0º). Anatomical angles were used to report subject-specific joint angles for the onset of reflex activity. The dynamometer angle corresponding with 90º elbow flexion was recorded in A/D units and used to reproduce the anatomical 90º elbow flexion position in subsequent evaluation sessions. Positioning was replicated at each session by recording the dynamometer and chair position settings for each participant.

Velocity-dependent reflex responses were tested by operating the dynamometer in passive mode under panel control. Each trial was comprised of four phases: i) 10 second static hold in elbow flexion; ii) passive elbow extension at criterion speed; iii) 5 second static hold in full extension; iv) passive return to elbow flexion at 30º/s. During all movement phases, participants were instructed to relax as the limb was moved through the full range of elbow motion by the dynamometer. Torque, position and EMG data were collected before and during passive elbow extension stretches. Passive stretches were delivered at five criterion speeds (i.e., 60º/s, 90º/s, 120º/s, 150º/s, 180º/s). After every third trial the test speed was incremented by 30º/s to obtain three trials at each criterion. Two additional trials were obtained at 10º/s to quantify passive joint torques. The reliability of both EMG and torque responses has been established for ramp-and-hold stretches obtained using this paradigm and range of speeds [41].

### Data analysis

#### Neuromechanical assessments

Torque, position and EMG were analyzed using MATLAB (Version 6.5.0)^d^. The torque and position signals were digitally lowpass filtered (20 Hz cutoff, zero-phase shift, 1^st^-order Butterworth filter). Velocity was determined by calculating the derivative of the filtered position signal. This calculated signal was subsequently digitally lowpass filtered at 20 Hz. Maximal isometric joint torque (MVIC), agonist EMG at MVIC, and peak power were evaluated for the five movements listed above. Muscle length and joint position effects were controlled by defining a 15º window centered at the optimal position^e^ for each joint action. Isometric, concentric and eccentric torque, velocity and EMG were evaluated over this range. Power was calculated as the product of torque and velocity within this window. Peak power was extracted from the condition (i.e., 30, 75 or 120º/s) producing the highest value. Neuromuscular activation was evaluated by determining the EMG amplitude during MVIC. Raw EMG signals were gain-corrected, filtered (10-200 Hz bandpass, zero-phase shift, 1^st^-order Butterworth filter), and the RMS average calculated over the same position window as torque [6].

#### Stretch reflexes

**Figure 4:**
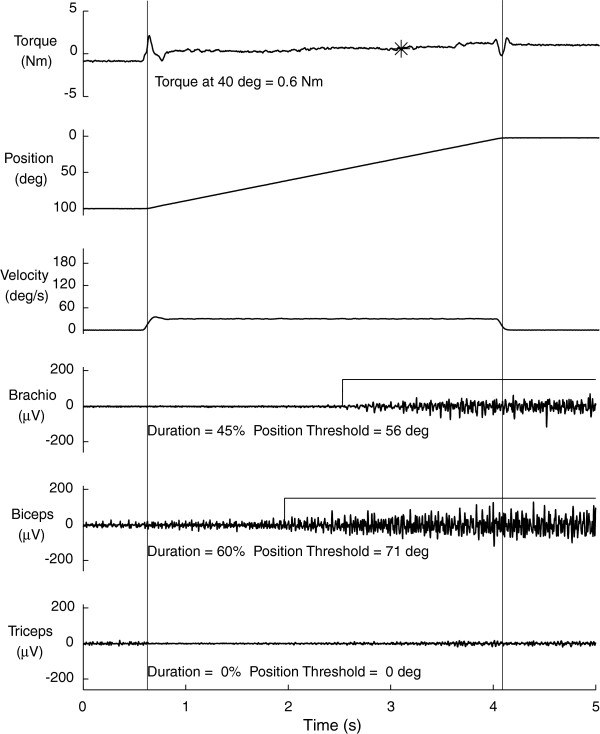
**Elbow stretch reflex responses.** Exemplary data from passive elbow stretches as described in methods. Top three panels illustrate torque, position and velocity, respectively, and bottom three panels, EMG from brachioradialis, biceps brachii, and triceps brachii, respectively. Vertical cursors mark trial onset and offset. Position reflects flexion at start (100º) and extension at end (0º). Velocity is constant over the period of passive stretch. Passive torque was measured at 40º elbow flexion for all individuals (noted by asterisk on top panel), which falls in the mid-range of joint position. Horizontal lines overlaid on brachioradialis and biceps EMG denote muscle activity “on” period. The position onset and duration of EMG activity were determined for each individual trial. Improvement in reflex modulation (e.g., reduced hyperreflexia) would reveal a reflex position threshold in a more extended position corresponding with lower values.

The slow (10º/s) passive torque response at each position was subtracted from the torque measured during stretches imposed at all speeds. Raw EMG signals were gain-corrected, filtered (200 Hz lowpass, zero-phase shift, 1^st^ order Butterworth filter), demeaned and rectified. EMG was evaluated as the mean amplitude calculated over a 100 ms sliding window. For each trial, EMG was defined as active when the mean amplitude exceeded threshold (i.e., mean baseline, resting EMG plus 2.5 standard deviations [43] (Figure 4). To assure analysis of only passive stretches, trials with EMG activity present within 200 ms of movement onset were not analyzed.

The processed EMG data were used to obtain three criteria (illustrated in Figure 4) indicative of stretch reflex modulation:

1. EMG Burst Duration – percentage of the movement time (MT) during which EMG activity was present.

2. Position Threshold – joint angle, expressed in degrees of elbow flexion, at which EMG activity was first identified. If the EMG activity was absent for the entire imposed stretch, the position threshold was reported as 0º, corresponding to full extension.

3. Burst Amount – mean EMG amplitude when the muscle was determined to be active minus baseline resting activity.

4. Torque – average torque calculated over a 100 ms window centered at 40 degrees of elbow flexion. Only trials in which the torque was 0.05 Nm greater than the slow passive torque (i.e., 10º/s) were considered in the analysis. Using this criterion, valid torques were not obtained at any speed for one participant at the post-treatment evaluation, and two participants at the retention period, thus their data were excluded from this analysis reducing the data set to 16 of 19 participants.

### Statistical analysis

#### Clinical assessments

Data were tested for normality using the D’Agostino & Pearson Omnibus normality test and found to be normally distributed. Baseline equivalence between treatment orders was confirmed using unpaired t-tests for between-group comparisons of clinical data. Three sets of comparisons were performed: the first two evaluated intervention-related changes between FTP and HYBRID, while the third tested for an effect of treatment order. The full set of comparisons included:

1) the primary treatment effect - evaluated by comparing change scores following treatment block1 (i.e., FTP vs. HYBRID);

2) block, or period, effect – evaluated by comparing the difference in magnitude of block1 and block2 change scores calculated within each treatment order (i.e., Order A: (HYBRID – FTP) vs. Order B: (FTP – HYBRID). Equivalent effects between interventions would yield a non-significant difference between treatment orders because differences in change scores between blocks would reveal a potential period effect. However, a significant, non-zero difference between orders A and B would occur in the presence of differential treatment effects for FTP and HYBRID [26].

3) The effect of treatment order – evaluated by comparing the overall change between baseline and completion of the second treatment block (i.e., sum of block1 and block2 change scores for each group (Order A vs. Order B).

Retention effects were assessed as differences between baseline and 6-month follow up. Missing data that resulted if participants were lost to follow up were treated using the last value carried forward [44].

To determine the scale of intervention-related differences, effect sizes were calculated using the difference between the means of the two interventions (FTP vs. HYBRID) divided by the common standard deviation (SD) at study baseline. Effect sizes were interpreted using benchmarks established by Cohen [45] where 0.2 is indicative of small, 0.5 medium, and ≥0.8 large effect sizes.

The primary outcome (WMFT-FAS) was evaluated using independent samples t-tests to test the hypothesis that improvements following HYBRID would exceed those in response to FTP.

Secondary clinical outcomes were evaluated by establishing the minimally important difference (MID) for each measure and testing for sample proportions achieving the MID. The MID is a distribution-based measurement approach [46] for determining clinically relevant change, defined as one-half of the standard deviation observed at baseline [47]. Differences between treatments (i.e., FTP vs. HYBRID) were probed using Chi-square analysis, and where appropriate Fisher’s Exact test, to test for the proportion of the study sample that produced the relevant MID.

#### Neuromechanical assessments

Torque and EMG data were tested using mixed-model repeated-measures ANOVA (RM-ANOVA) with main effects of treatment order (group), treatment and joint action. Tukey’s HSD test was used for post-hoc testing to identify the location of significant effects.

#### Stretch reflex assessments

To account for inter-subject variability all measures were evaluated as change scores relative to baseline. The magnitude of change in EMG responses to imposed stretch was assessed for both significant within-group changes relative to baseline and for between-group differences. Within each group, single factor t-tests were used to determine if the mean change, pooled across speeds, differed significantly from no change. Between-group differences were assessed using RM-ANOVA.

Statistical analysis was performed using SAS Release 6.12 (reflex data) or JMP (Version 9.0)^f.^ Unless otherwise specified, statistical significance was established as *p* < 0.05.

## Results

### Clinical assessments

#### Primary outcome

**Figure 5:**
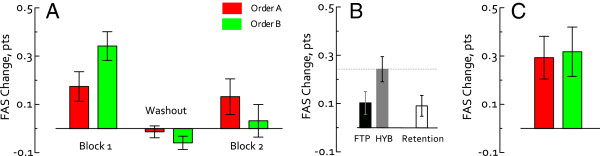
**WMFT FAS change scores.** The primary outcome was analyzed by evaluating change scores (post-pre). **Panel A**. FAS change scores plotted by treatment block. Participants in treatment Order A (red) received FTP first. Improvements in the FAS score were similar between blocks 1 and 2. Participants in treatment Order B (green) received HYBRID first. Improvements in the FAS score were larger in Block 1 (HYBRID) than Block 2 (FTP). Negligible changes were detected following the washout period. **Panel B**. Change scores pooled across treatment blocks for FTP and HYBRID reveal significantly greater improvements following HYBRID (gray) vs. FTP (black). **Panel C**. Overall differences were not revealed between treatment orders (Order A-red, Order B-green) following both treatment blocks (i.e., post-block2 – baseline) and the intervening washout period. At the 6-month follow up, additional, small changes in FAS scores were detected (**Panel B**, white bar); effects were similar between Order A and Order B.

Our primary aim was to determine whether power training contributes to functional improvements in the hemiparetic upper-extremity. For the primary outcome (WMFT-FAS), improvements significantly different from zero were revealed following treatment block1 following both FTP and HYBRID (p < .05). These differences were significantly greater following HYBRID (mean 0.34 ± 0.06(S.E.)) as compared to FTP (mean 0.17 ± 0.06(S.E.)) (p = .03). Figure 5, Panel A). Testing for a period effect revealed greater improvements following HYBRID vs. FTP (p = .02) (Figure 5, Panel B) regardless of where they occurred in the treatment order (p = .02). Overall differences due to treatment order were not revealed (e.g., Order A, FTP-first (mean 0.29 ± 0.09(S.E.)) vs. Order B, HYBRID-first (mean 0.32 ± 0.10 (S.E.)), *p* = .43) (Figure 5, Panel C). FAS change scores improved further (mean increase: 0.09 ± .04 (S.E.) points) during the 6-month follow up period. While the magnitude of change was small, this improvement was significantly different from zero (*p* = .03), indicating both retention of treatment effects and advancement of these functional improvements over the 6-month follow up interval. Differences between Order A and Order B were not revealed at 6-month follow up (*p* > .05).

#### Secondary outcomes

Improvements were detected in both the total and shoulder-elbow portions of the upper-extremity Fugl-Meyer score, however no intervention-related differences were revealed in the proportion of participants who achieved the MID immediately post-treatment (Table 2). At 6-months, the MID for the shoulder-elbow sub-score was achieved by 53% of all participants (*p* = .04) indicating that treatment-related effects were both retained and advanced during the retention period. No significant changes were revealed on the combined shoulder-elbow Ashworth score at either the post-intervention or 6-month retention evaluation (p > .05). A significantly greater proportion of participants (51% vs. 39%) produced the MID of two points or more on the FIM following HYBRID (*p* = .05). These positive changes were observed in 69% of participants at 6-months (*p* = .05). Mean change scores calculated for each of the clinical assessments are reported in Table 2.

**Table 2:** **Clinical results**: **post**-**intervention and retention effects**

**Intervention effects**				
**Measure**	**FTP**	**HYBRID**	**E.S.**	
**Primary Outcome**				
Wolf Motor Function Test (FAS, Range 0-5 pts)	0.10	0.24	0.50	*
	(SE 0.05)	(SE 0.05)		
*Secondary Outcomes*				
UE Fugl-Meyer Motor Score (66 pts)	2.94	2.89	*0*.*2*	*n*.*s*.
MID ≥5	(SE 0.72)	(SE 0.79)		
Fugl-Meyer Shoulder-Elbow Score (30 pts)	1.83	0.79	*0*.*87*	*n*.*s*.
MID ≥3	(SE 0.40)	(SE 0.38)		
Ashworth Scale (shoulder + elbow, 8 pts)	-0.16	-0.11	*0*.*07*	n.s.
MID ≥1	(SE 0.24)	(SE 0.24)		
Functional Independence Measure (91 pts)	0.67	2.21	*0*.*81*	*
MID ≥2	(SE 0.63)	(SE 0.61)		
**Retention Effects*(6***-***month Follow Up)***			**E.S.**	
**Measure**				
**Primary Outcome**				
Wolf Motor Function Test (FAS, Range 0-5 pts)	0.10		0.65	*
	(SE 0.05)			
*Secondary Outcomes*				
UE Fugl-Meyer Motor Score (66 pts)	7.2		*3*.*09*	*
MID ≥5	(SE 1.55)			
Fugl-Meyer Shoulder-Elbow Score (30 pts)	2.82		*2*.*35*	*
MID ≥3	(SE 0.51)			
Ashworth Scale (shoulder + elbow, 8 pts)	-0.21		*0*.*28*	*n*.*s*.
MID ≥1	(SE 0.24)			
Functional Independence Measure (91 pts)	3.6		*1*.*89*	*
MID ≥2	(SE 1.21)			

Data are presented as mean (SE) change scores and reflect mean changes due to the treatment specified (i.e, Post-Pre for block involving FTP or HYBRID). Retention effects at 6-month follow up reflect change scores from the follow up period (i.e., 6-months–post-treatment).

Unless otherwise noted, statistical significance was set at *p* < .*05* (*denoted as* *) and reflects significant differences between treatments. Secondary outcomes were tested for differences in the proportion of participants demonstrating an MID post-treatment. Effect sizes (ES) reflect between-group differences. 6-month follow up data reflect retention and/or advancement of primary treatment effects. Due to the cross-over design effects reflect overall differences relative to baseline.

### Neuromechanical assessments

#### Isometric joint torque

**Figure 6:**
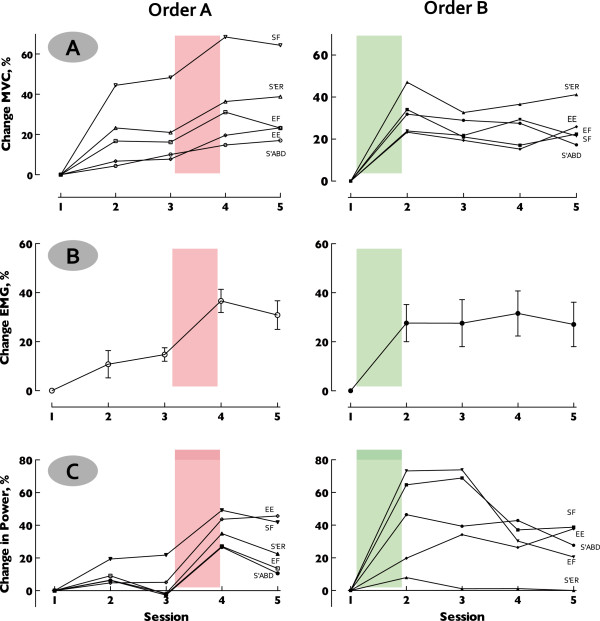
**Neuromechanical responses.** Data presented as % change relative to baseline to illustrate the evolution of responses over both treatment blocks and the 6-month retention period. Similar patterns are revealed across all measures: isometric joint torques (**Panel A**), EMG (**Panel B**) and joint power (**Panel C**) indicating a robust physiological response to the HYBRID intervention. Left column plots results for treatment Order A (FTP first) highlighting treatment block 2 when HYRBID intervention was delivered. Right column plots results for treatment Order B (HYBRID first) highlighting block 1 when HYBRID intervention was delivered. **Panel A**. Isometric joint torques, plotted by individual movements. **Panel B**. Agonist muscle EMG at maximal voluntary isometric contraction. Data collapsed across movements reveal a distinct pattern of increased EMG activation following the HYBRID intervention, independently of where it occurred in the treatment order. **Panel C**. Peak joint power by individual movement. Pattern of response is consistent across movements, although magnitude of change varies. Note loss of power following treatment block 2 (FTP) in Order B, likely resulting from lower intensity of activities in the FTP.

The magnitude of change in isometric joint torques was similar among the five joint actions tested (*p* = .53) (range 11.03% (±9.6) – 28.4% (±10.0)). A significant effect of treatment revealed greater increases in isometric joint torque following HYBRID (28.17% (±3.9)) than FTP (12.5% (±4.2)) (*p* < .0001). Changes in isometric joint torque relative to baseline are illustrated by treatment order and individual joint action in Figure 6, Panel A. No interactions of group (treatment order) or joint action were revealed (*p* > .05).

#### EMG at maximal voluntary isometric contraction

Similar to effects reported for isometric joint torque, the magnitude of change in agonist muscle EMG during MVIC was similar among the joint actions tested (*p* > .05) (range: 10.79% (±5.5) – 36.63% (±4.7)). A significant effect of treatment revealed greater increases following HYBRID (24.74% (±6.2)) as compared to FTP (7.34% (±7.4)) (*p* < .0001). Changes in EMG at MVIC, relative to baseline, are illustrated by treatment order and joint action in Figure 6, Panel B. No interactions of group (treatment order) or joint action were revealed (*p* > .05).

#### Joint power

Changes in peak power for each movement paralleled effects revealed in isometric joint torque and EMG at MVIC, described above. As would be expected, significant differences in peak power were revealed between joint actions (shoulder external rotation (726.5 W) < elbow extension (969.7 W) = shoulder abduction (1109.3 W) = shoulder flexion (1162.0 W) < elbow flexion (1688.7 W)) (*p* < .0001).

A significant effect of treatment revealed markedly greater increases in joint power following HYBRID (36.66% (±11.6)) as compared to FTP (-7.86% (±3.5)) (*p* < .0001). Changes in joint power relative to baseline are illustrated by treatment order and individual joint action in Figure 6, Panel C. Negative changes, indicating loss of joint power following FTP, result from small changes revealed in treatment Order A (mean 9.22% (±2.6)) combined with relative loss of power revealed in treatment Order B (-19.57% (±8.6)) when FTP was the second intervention. Importantly, for treatment Order B joint power remained elevated relative to baseline (24.65% (±4.3)) following FTP. No interaction effects of group or treatment and movement were revealed.

**Figure 7:**
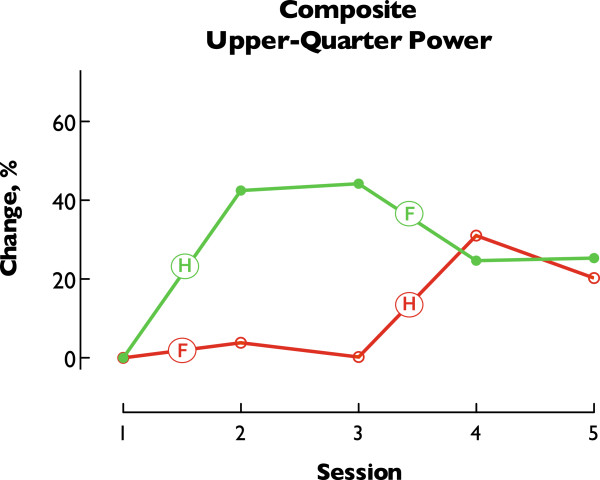
**Composite Upper**-**quarter joint power.** Peak power from all movements was collapsed within each treatment order (Order A–Red, Order B–Green) creating a composite representative of upper-quarter performance. Data are expressed as % change relative to baseline and demonstrate the evolution of response over all phases of the study. Labels note relevant treatment (F: FTP, H: HYBRID). Of note, the magnitude of improvements in response to HYBRID was similar regardless of when the HYBRID intervention was delivered. This result illustrates the strength of using a crossover design to differentiate treatment effects. Overall improvements following both treatment blocks (Session 4) reveal similar changes relative to baseline. Note that increased upper-quarter power is similar between Sessions 4 and 5 indicating retention of improvements at 6-months post-intervention.

Following completion of both intervention blocks (e.g., Session 4), joint power was significantly increased relative to baseline with similar improvements revealed in both treatment orders (Order A: 31.06% (±9.1), Order B: 24.65% (±4.3)). At the 6-month follow up evaluation (e.g., Session 5) increased joint power was retained in both groups (Order A: 20.24% (±6.4), Order B: 25.36% (±8.0)). The magnitude of changes in joint power following HYBRID did not differ statistically between Order A (30.84% (±9.8)) and Order B (42.48% (±9.1)) (*p* > .05), thus revealing the specific effect of the HYBRID intervention rather than generalized exposure to therapeutic intervention. Overall treatment and retention effects are illustrated in Figure 7.

#### Stretch reflexes

Data were obtained from only the first block of the crossover, thus results reflect effects of only a single intervention (i.e., FTP or HYBRID). Brachioradialis responses demonstrated similar patterns at reduced magnitude and triceps responses were negligible. Results and discussion presented here thus focus on the biceps brachii responses. Usable data were not available all participants for all evaluations, thus the number included is stated for each analysis.

**Figure 8:**
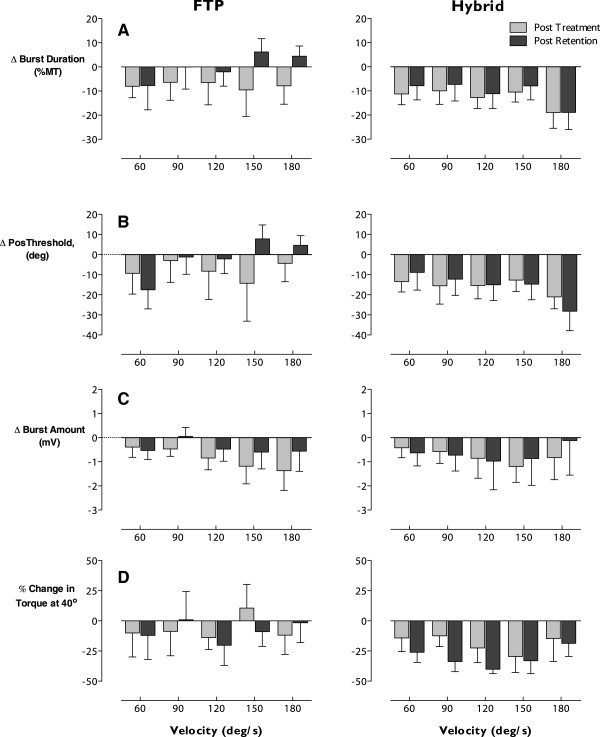
**Adaptations in stretch reflex responses.** Velocity-dependent responses to passive elbow stretch expressed as change scores relative to baseline for post-treatment (gray) and post-washout (black). Data are presented for the first block of the crossover, thus reflect response to a single treatment, FTP (left column) or HYBRID (right column). Negative values signify improvement (i.e., reduced EMG activity (**Panel A**), position threshold in greater elbow extension (**Panel B**), reduced passive torque (**Panel D**)). Positive values would indicate exacerbation of stretch-induced activity. Systematic, velocity-dependent improvements are revealed across parameters following HYBRID. While some improvements are noted following FTP, these are less consistent and not well retained over the 4-week washout. Results for the burst amount variable (**Panel C**) did not reach statistical significance, but are included to illustrate the consistent effect. Improvements in passive torque were greater and reached statistical significance following the washout. Taken together these results are consistent with the differential rate of neural (early) and muscular (later) adaptations.

Adaptations in biceps stretch reflex activity were revealed as mean negative change in response to passive elbow extensions as measured by EMG variables burst duration, position threshold and burst intensity indicating: shorter burst duration, reflex onset at a more extended position and reduced EMG intensity, respectively. Changes observed following intervention are illustrated in Figure 8.

#### Burst duration

Following intervention, the mean duration of biceps activity was reduced following both FTP and HYBRID, although this change differed significantly from zero only following HYBRID (*p* = .03). Following FTP, only 4/6 participants demonstrated reduced burst duration (mean change -7.6% MT (SE 2.9)), which did not differ statistically from zero (*p* > .10). In contrast, following HYBRID 8/9 participants revealed a significantly reduced burst duration that averaged -12.5% (SE 1.5) MT across speeds. At retention, 3/7 FTP participants and 6/9 HYBRID participants maintained this change to produce mean group changes of -0.2% MT (SE 2.3) (*p* > .10) and -10.8% MT (SE 1.6) (*p* = .06), respectively. Group data for each speed and evaluation are presented in Figure 8, Panel A.

RM-ANOVA was used to test for effects at each criterion speed. When data from all tested criterion speeds (i.e., 60º/s – 180º/s) were included, between-group differences failed to reach statistical significance following the retention period. However, the results suggested the presence of an interaction between the group and velocity factors that approached statistical significance (F_(4,54)_ = 2.15, *p* = .087). Coupled with our prior investigation that revealed greater stability of reflex responses at higher speeds of stretch [41], this observation motivated a secondary analysis. Responses at criterion speeds ≥120º/s revealed a greater reduction in burst duration in response to HYBRID which reached statistical significance (F_(1,14)_ = 4.74, *p* < .05) following the retention period.

#### Position threshold

The pattern of changes in the position threshold was similar to that observed in the burst duration. Both groups demonstrated improvements, but mean differences post-intervention reached statistical significance and were retained only following HYBRID. Negative change scores in position threshold indicate later onset of biceps EMG activity, at a more extended position. Post-intervention, 4/6 FTP participants and 8/9 HYBRID participants demonstrated a decreased position threshold at most speeds. Following the retention period, only 3/7 FTP whereas 8/9 HYBRID participants demonstrated this improvement (Figure 8, Panel B). Collapsed across speeds the mean change following FTP was -7.8 degrees (SE 4.6) and -1.7 degrees (SE 2.2) post-intervention and post-retention, respectively. Neither change differed from zero (*p* > .10). Following HYBRID, corresponding change scores reached statistical significance and averaged -16.5 degrees (SE 1.9) post-intervention and -15.8 degrees (SE 1.8) post-retention (*p* = .02 after both periods).

Consistent with the results for burst duration, when data from all speeds were tested using RM-ANOVA, significant between-group differences were not revealed. However, analysis of this variable also suggested an interaction effect of group and velocity (F_(4,54)_ = 2.53, *p* = .051). Secondary analysis of speeds ≥120º/s revealed greater changes in the position threshold following HYBRID than FTP that reached statistical significance following the retention period (F_(1,14)_ = 6.03, *p* < .05).

#### Burst amount

As with the other parameters presented, negative changes in burst intensity indicate reduced stretch-induced biceps activity and therefore represent improvements (Figure 8, Panel C). The majority of participants demonstrated improvements following intervention (4/6 FTP, 6/9 HYBRID). Collapsed across speeds these improvements represented a mean change of -0.85 mV (SE 0.22) following FTP and -0.88 mV (SE 0.22) following HYBRID. Following the retention period, only 3/7 FTP participants demonstrated these improvements with a mean change of -0.45 mV (SE 0.20). However, 5/9 HYBRID participants retained improvements with a mean change of -0.78 mV (SE 0.31). While none of these changes differed significantly from zero (*p* > .10), the pattern revealed is consistent with that observed in the burst duration and position threshold variables, thus these data are included for sake of completeness. Improvements occurred in response to both interventions but at follow up were retained only in individuals who received HYBRID.

#### Torque responses

No consistent changes in the passive torque response were revealed following FTP. Collapsed across speeds, mean changes in passive torque following FTP were -6.9% (SE 4.7), and -10.1% (SE 6.4) following intervention and retention, respectively, and failed to reach statistical significance (*p* > .10). Passive torque was reduced in 4/6 individuals following FTP and 6/7 participants following the retention period. While these proportions suggest greater improvements following the retention period, mean changes at each speed expressed as a percentage of baseline torque (Figure 8, Panel D, left) reveal large variability. In particular, one individual produced large increases in torque.

In contrast, following HYBRID 7/8 participants demonstrated a reduction in the resistance to imposed stretches corresponding to a group mean of -15.3% (SE 4.3). This effect also failed to reach statistical significance (*p* > .10). However, following the retention period, passive torque was reduced in all 7/7 participants. Importantly, not only were the reductions revealed following the HYBRID intervention retained, but the magnitude was greater following the retention period reaching -30.3% (SE 1.4) which differed significantly from zero (*p* < .001). Thus, the HYBRID intervention appeared to produce systematic changes in passive torque across speeds (Figure 8, Panel D, right) of greater magnitude to those detected following FTP. However, due to large inter-subject variability statistically significant differences were revealed only within each group.

## Discussion

This study investigated the feasibility, safety and efficacy of upper-extremity power training in persons post-stroke. Our main finding is that inclusion of power training (i.e., dynamic, high-intensity resistance training) in a program of upper-extremity rehabilitation is feasible, without negative consequences including either musculoskeletal compromise or exacerbation of spasticity. Functional recovery, as documented by the WMFT-FAS and other clinical indicators, was greater following HYBRID than FTP. Intervention-related effects were both retained and, in some cases, advanced during a 6-month retention period. To our knowledge, this is the first study demonstrating advancement of intervention-related improvements over a 6-month period of no additional intervention.

Several novel aspects of the intervention reported here likely contribute to our positive results: 1) high-intensity workloads with progression to advance the challenge over the course of the intervention [48]; 2) dynamic contractions that challenged the impaired nervous system to increase movement speed and muscle power; 3) presentation of eccentric contractions which – a) increases the absolute magnitude of the training stimulus, b) involves alternative neural strategies for execution, c) requires force production throughout the full range of motion and therefore facilitate reacquisition of this critical neural mechanism of force production.

### Relationship of findings to current research results

Other studies have compared strengthening and task practice for persons post-stroke in the sub-acute [49] and chronic [50] periods of recovery with conclusions of both favoring functional task practice. Careful examination of the methods and training parameters, however, reveals that the training approach used in the present study differed considerably. Among those previous studies, the first based strengthening on functional activities performed with either increased resistance or repetitions, while the second utilized an independent home-based program of limited scope and intensity. Most notably, therapeutic activities in both studies were not graded relative to maximal capacity and algorithms for progressive challenge of resistance training were not evident. A third study utilized a uniplanar robot to deliver a high volume of resisted upper-extremity movements, all performed in the transverse plane at table top height [51]. Similar to the outcomes of the activity-based functional therapies described above, resisted and non-resisted robotic therapy appeared equally effective. However, the peak resistance level presented in the entire six-week robotic protocol was 28 N (~6.3# or 2.9 kg) and an algorithm for systematic progression of the resistive load was not evident. Using grip force as a proxy for upper-extremity strength, normative values for MVIC grip force average 236 N and 383 N for women and men, respectively, aged 60-69 [52] indicating that the resistance used in this robotic study involved only 7-10% of maximal capacity. These three studies each concluded no benefit of strengthening for improving function in the hemiparetic upper-extremity. Yet, in all three cases the resistance intervention may have lacked sufficient contrast to the alternative task-specific practice approach. More importantly, in all three cases the intensity of the resistance was most likely insufficient to represent an overload stimulus [53], which therefore readily explains the failure to produce meaningful effects on either strength or function. Because the current study involved dynamic contractions, direct comparison to the resistance levels used in the three earlier studies is not possible. As explained in the description of the therapeutic interventions (Figure 3), the training prescription in the current study differed from previously conducted studies in three ways: 1) resistance exercise targeted contractions at specific velocities, 2) intensity of the resistance required a high level of the participant’s maximal capacity and 3) work load was systematically progressed over the course of the intervention.

In contrast, a recent study utilized a robotic-type device that offered both static resistance (i.e., isometric) and repetitive arm movements at preset constant velocities (i.e., isovelocity) that required production of a minimum threshold force throughout the full range of motion [54]. Eight weeks of training (24 sessions) using this combination of parameters (i.e., threshold force throughout the movement, dynamic contractions, systematic repetition) in persons six or more months post-stroke produced increases in grip and isometric shoulder strength ranging from 22–62% and modest gains on the UE Fugl-Meyer assessment, both outcomes comparable to those revealed in the present study. Perhaps more remarkable were significant improvements in critical parameters of reaching including: movement speed, time-to-peak velocity, minimum jerk and inter-joint coordination suggesting that repetitive training on the basis of key biomechanical parameters facilitates improved coordination of multi-segmental upper-extremity movements.

### Does improved strength relate to improved function?

Weakness has long been recognized as a prominent characteristic of post-stroke hemiparesis, yet the relationship between increased strength and improved function has been elusive. Despite evidence of beneficial effects of strengthening, evidence to support concurrent effects on functional motor performance remains equivocal [55,56]. Accordingly, prevailing clinical perspectives assert that remediation of weakness is a problem distinct from restoration of function and task-specific practice is requisite to promote improved functional performance [49,56]. Moreover, there is strong evidence to suggest that repetitive task practice drives neural plasticity at the supraspinal level [57,58]. Given these assertions the results of the present study are novel. HYBRID produced significant improvements not only in isometric strength, neuromotor activation and power production, but clinical parameters of impairment and functional activities. To our knowledge, only two other studies [21,23], have reported improvement in upper-extremity function following resistance training. While we recognize that the HYBRID intervention combined functional task practice and power training, the results reveal larger effects on all measures compared to functional task practice alone. Thus, it appears that functional outcomes are improved by directly addressing the weakness component of post-stroke hemiparesis.

The majority of studies pertaining to persons post-stroke characterize weakness using isometric force measurements and from these data it has been concluded that improved strength does not contribute to improved function. Because functional task performance is dynamic, characterization of muscle performance under dynamic conditions is more relevant to understanding functional motor impairment. Indeed, intervention-related increases in dynamic torque generation have been revealed in conjunction with absence of improvements in isometric force [21]. Power represents the capacity to generate force over time (i.e., in a moving joint [48]). Quantification of a dynamic muscle performance parameter, such as power, may thus reveal the elusive link between strength and enhanced functional performance relevant to profoundly motor compromised populations such as post-stroke hemiparesis.

A stronger relationship has been demonstrated between power and function than between strength and function in older adults [59,60]. The contribution of neuromotor control mechanisms to this relationship is unmistakable. For example, reduced power production in mobility-limited elders is strongly associated with the rate of EMG production [61]. Conversely, older adults who maintain competitive fitness for power lifting retain maximal motor unit firing rates at levels comparable to healthy young individuals [62]. High-velocity and/or explosive training increases neuromuscular and mechanical power to a greater extent than strength training and is associated with improved performance on functional tasks [59,63]. Leveraging these findings we questioned whether the obvious manifestations of neuromotor impairment following stroke would respond similarly to older adults without neuropathology. Additional work in our laboratory, separate from this current study, has demonstrated that upper-extremity power training in isolation (i.e., not combined with FTP) is equally, if not more, effective than FTP for promoting recovery of functional upper-extremity movements [23].

### Strength and activation changes

The early phase (i.e., 2-6 weeks) of resistance training is known to produce neural adaptations which influence the magnitude and organization of motor output (e.g., “central motor drive”) and may include: improvements in cortical excitability, alterations in motor unit recruitment threshold, changes in motor unit firing patterns (e.g., increased recruitment, rate coding, presence of doublets, motor unit synchronization, etc.) [64-67] and alteration in the patterns of force production including an increased rate of force production [68]. Both the magnitude and time course of increased isometric strength, EMG at MVIC, and joint power in response to HYBRID are consistent with such neural adaptations [66].

Recent work documents both increased corticospinal excitability and marked reduction of GABA-mediated short intracortical inhibition (SICI) following 4 weeks of dynamic, high-load resistance training [69]. While this work provides clear evidence of functional changes in the strength of corticospinal projections following resistance training, reduced SICI may be more relevant to the current study and individuals post-stroke. Corticomotor drive results from the net balance of excitatory and inhibitory influences integrated by the intra-cortical circuits [70]. Reduced SICI reveals reduced inhibition, resulting from unmasking of silent synapses (e.g., disinhibition) and, potentially, synaptic plasticity at the cortical level [58,71]. Excessive inhibition of the ipsilesional hemisphere is recognized following stroke and restoration of the balance of cortical excitability between hemispheres is now acknowledged as a target for motor rehabilitation [72]. This recent demonstration of cortical disinhibition in response to dynamic, high-load resistance training suggests potential mechanisms mediating the positive neuromechanical and functional outcomes demonstrated in the present study, which can be systematically investigated in future research.

### High-exertion activity does not exacerbate spasticity

Our results also reveal concurrent improvements in biceps brachii stretch reflex modulation and upper-extremity functional use in response to HYBRID. While clinical assessment using the Ashworth Scale revealed no significant changes following either FTP or HYBRID, both stretch reflex modulation (e.g., hyperreflexia) and passive torque responses (e.g., hypertonia) were significantly improved following HYBRID. Comparable effects were not revealed following FTP.

We hypothesized that high-intensity activity would not exacerbate spasticity. Unexpectedly, our findings demonstrate that high-intensity motor activity actually induces positive adaptations in reflex modulation that are retained in the absence of additional intervention. Previous work investigating the mechanisms of hyperreflexia has provided evidence for: increased/abnormal motoneuron excitability [73]; increases in activation of dendritic persistent inward currents [74-76]; decreased presynaptic inhibition [77]; diffuse changes at the level of spinal circuitry affecting responses in multiple muscles [78-80], and aberrant depolarizing synaptic drive [81]. Reductions in aberrant activity, including systematic changes in the onset threshold of reflex activity as observed following HYBRID, can thus be considered positive adaptations in the direction of normal stretch reflex activity. The behavioral manifestations of neural recovery undoubtedly involve the integration of adaptations throughout the neuraxis. When studied concurrently with clinical and functional performance, reflex responses provide a means to monitor these multi-factorial physiological adaptations.

### Active control

In the present study the experimental, HYBRID, intervention was compared directly to an active control intervention (FTP). The functional task practice program was developed according to principles guiding current clinical practice [82] and afforded dose-equivalent matching for treatment time, time on task, and practitioner exposure. Repetitive task practice is argued as the intervention approach of choice for driving functional reorganization of the nervous system post-stroke [24,49,56]. While intervention-related effects were indeed observed in response to the control intervention, the experimental intervention produced both larger changes and a larger proportion of participants producing clinically significant improvements. In contrast to many investigations of rehabilitation efficacy [24,83,84], our approach was to determine whether the experimental intervention would produce greater effects than a standardized treatment developed to meet the putative parameters of current clinical practice. In so doing, we anticipated that the control intervention would reveal treatment-related gains.

### Crossover design

Our use of a crossover design enabled us to monitor responses to both interventions in the same individuals strengthening our findings regarding differential treatment effects between HYBRID and FTP. Crossover designs offer two clear advantages. First, the influence of confounding covariates and heterogeneity between individuals is reduced because each participant serves as his/her own control. It can be expected that an intervention will produce large and small responses among individuals and similarly, that individuals may be high and low responders. Thus, the crossover can detect differential responses to therapies, should they exist. Second, optimal crossover designs are statistically efficient, thus require fewer subjects [26].

Crossover studies also present challenges, two of which are the potential of order effects and the potential of carry-over between treatments. It is possible that the order in which treatments are administered will affect the outcome [85]. In the case of rehabilitation, this outcome may be genuine in that one treatment order is more efficacious or may result from a variety of influences. Clinical assessments typically used in rehabilitation are not optimally sensitive or responsive to change and thus are prone to ceiling and floor effects. Compounding these problems of clinical assessment there may be a learning effect or physiological conditioning effect in response to active therapy following a period of relatively sedentary lifestyle. Taken together, these circumstantial influences may contribute to greater responses to the first treatment, regardless of which treatment occurs first. A second concern when using a crossover design is the potential of carry-over between treatments. Carry-over effects are of particular concern in the case of rehabilitation, or exercise, where the intent is to induce persistent changes. In practice, carry-over effects can be avoided with a sufficiently long washout period between treatments. In the worst case, if treatment effects are non-specific and retained through a washout period, a crossover design would yield the obvious result – more therapy is better. In the best case, a crossover design can reveal differential effects of intervention and may suggest order effects that would optimize the ordering of activities in rehabilitation [23]. In the present study, the differential effects of FTP and HYBRID can be appreciated across all levels of measurement, clinical, neuromechanical and neurophysiological. While period effects are suggested in some measures (e.g., Figures 5 &6), they were not consistently revealed and thus contrast with our recent work [23]. The interventions in the present study shared common elements (i.e., HYBRID involved an abbreviated program of FTP), thus the distinction of ordering may be less clear than when the interventions are contrasting. Regardless, distinct differences in the magnitude of improvements were revealed favoring the HYBRID intervention, which incorporated power training.

### FAS

Given the underlying rationale of objectively assessing movement function with a standardized battery of timed tasks, one might question the choice of the observational, FAS component of the WMFT. The psychometric properties of the WMFT including validity, reliability and discriminant capacity have been established [38]. Consideration of the FAS may be an underappreciated aspect of this literature. Since early efforts, both validity and reliability of the FAS component have been tested and reported [37]. Furthermore, early stages of the ExCITE trial reported psychometrics of all aspects of the WMFT, including the FAS, across study sites [36]. The FAS is equally reliable as the timed portion, and shows a significant negative correlation with performance time [36]. The fundamental point of both these analyses and inclusion of the FAS as a component of the WMFT is that movement speed and quality of movement are interrelated. Work recently published from our lab [23] used the WMFT to assess recovery of upper-extremity motor function post-stroke. Similar to the current study, we sought to understand the differential effects of two treatment interventions. Of note, the WMFT(time) improved equally in response to both interventions, indicating global improvements in motor function. However, kinematics (3D motion capture) differentiated treatment effects between groups with substantial effect sizes, while effect sizes for WMFT(time) were small to negligible for differences between groups [23]. Given that the primary question in the current study was to differentiate treatment effects, we elected to report changes in the FAS score. While observational, the FAS score incorporates features of movement captured quantitatively with kinematics. Perhaps more importantly, it affords a measurement instrument readily available to the practicing clinician.

### Limitations

While results of the present study are encouraging, there are a number of limitations and future investigation is clearly warranted to elaborate these early findings. The small sample size limits both generalizability and the ability to better understand whether differential treatment effects occurred in higher and lower functioning participants. Further, although hand function is clearly a critical element driving use of the upper-extremity, this phase of our investigation targeted the shoulder and elbow for both strengthening and functional effects. Our intention was to determine the feasibility, safety and efficacy of performing such high-intensity activity in persons post-stroke. With these fundamental issues addressed we are able to refine the intervention for future investigation. All treatments were delivered by one physical therapist. Due to the interpersonal nature of rehabilitation practice, it is likely that an element of our results can be attributed to the positive experience participants enjoyed in receiving a substantial bout of one-on-one treatment from a therapist with whom they enjoyed a good rapport. In future work additional personnel will be involved in an effort to generalize our findings.

## Conclusions

This efficacy trial of combined functional task practice and power training produced positive, meaningful effects on both clinical and neuromechanical metrics of upper-extremity impairment and function that were both retained and advanced over a 6-month retention period. Importantly, no adverse events were noted and no deleterious consequences, including exacerbation of spasticity, resulted from the high-intensity effort.

## Endnotes

^a^Biodex, Medical Systems, Shirley, New York, 11967-4704 USA.

^b^MA-311, Motion Lab Systems, Baton Rouge, LA 70816 USA.

^c^Keithly Instruments, Inc., Cleveland, OH 44139 USA.

^d^The Mathworks, Inc., Natick, MA, 07160-2098 USA.

^e^Elbow flexion: 48-63º, Elbow extension: 67-82º, Shoulder flexion: 15-30º, Shoulder abduction: 33-48º, Shoulder external rotation: 3-18º. Positions for optimal torque production were identified during pilot testing.

^f^SAS Institute, Cary, NC 27513 USA.

## Abbreviations

EMG: Electromyography; FTP: Functional task practice; HYBRID: Combined power training and functional task practice; MID: Minimal important difference; MVIC: Maximal voluntary isometric contraction force; WMFT: Wolf Motor Function Test; ANOVA: Analysis of variance; SD: Standard deviation; ES: Effect size; MT: Movement Time.

## Competing interests

The authors declare that they have no competing interests.

## Authors’ contributions

CP conceived, designed and executed the study, conducted the statistical analyses and drafted the manuscript; EGC developed data analysis methods, performed data analysis and interpretation and contributed to drafting the manuscript; CAD contributed to data acquisition, data analysis and data interpretation; PSL conceived, designed and executed the study, developed data acquisition methods, participated in data analysis and interpretation. All authors read and approved the final manuscript.
